# Age-dependent progenitor switching shapes adult brown adipose tissue heterogeneity

**DOI:** 10.21203/rs.3.rs-8515565/v1

**Published:** 2026-01-21

**Authors:** Hai-Bin Ruan, Chenxin Gu, Zengdi Zhang, Shaolei Xiong, Jiawen Ma, Zuoxiao Shi, Zan Huang, Sunhye Shin, Aneesh Swaminathan, Ifrah Aden, Zahra Moazzami, Xiaoli Wu, Christina Camell, Yuwei Jiang

**Affiliations:** University of Minnesota; University of Minnesota; University of Minnesota; University of Illinois at Chicago; University of Minnesota; University of Illinois Chicago; Nanjing Agriculture University; Seoul Women’s University; University of Minnesota; University of Minnesota; SOUTH DAKOTA STATE UNIVERSITY; Harvard University; University of Minnesota; University of Illinois at Chicago

## Abstract

The ontogeny of brown adipose tissue (BAT) begins during embryogenesis and continues into the postnatal period and throughout adulthood. Distinct populations of adipose progenitor cells (APCs) have been identified to support BAT development and thermogenesis; however, the division of labor and temporal relationship between different APCs, particularly during adulthood and aging, remain undetermined. Here, we showed that Pdgfra+ APCs establish BAT in early life but have a limited contribution to brown adipogenesis in adult mice housed at room temperature. Using integrative single-cell analysis and lineage tracing, we identified a distinct population of Myl1-expressing cells that emerge in an age-dependent manner and function as committed BAT progenitors in adult and middle-aged mice. Myl1+ APC-derived brown adipocytes possess a unique molecular signature that links to more dependence on oxidative phosphorylation over glycolysis, thus contributing to heterogeneous metabolic activity in adult BAT. The ablation of Myl1+ descendants or the blockade of Myl1+ APC differentiation leads to BAT paucity and impaired glucose metabolism in adult mice. Collectively, our results support a progenitor switching model in which distinct APC populations control sequential brown adipogenesis and shape BAT heterogeneity.

## Introduction

Brown adipose tissue (BAT) is a thermogenic organ conserved in placental mammals, and its prevalence is associated with metabolic health in adult humans^1–3^. Upon activation by cold, brown adipocytes consume large amount of glucose, fatty acids, and other fuels to generate heat via UCP1-dependent mitochondrial uncoupling and ATP-consuming futile cycles^4,5^. As an endocrine organ, BAT also secretes “batokines” and “lipokines” to regulate systemic homeostasis^6,7^. For these reasons, BAT transplantation and cell-based engineering have been shown to improve glucose metabolism and confer physiological benefits in preclinical models^7,8^. However, the prevalence of human BAT progressively decreases with age^9–11^, challenging the thermogenic capacity and therapeutical potential of BAT in middle-aged and elderly individuals. Mechanistic insights into the impact of age on BAT are urgently needed but significantly lacking.

Brown adipocytes share the same embryonic origins as myogenic cells^12^. The dermomyotome marked by *Myf5* gives rise to dorsal BAT, including the interscapular depot^13–18^, while *Tbx1^+^* progenitors within the pharyngeal mesoderm develop into supraclavicular BAT^19^. Single-cell RNA sequencing (scRNA-seq) and lineage tracing experiments have defined distinct populations of adipose progenitor cells (APCs) that control BAT development and homeostasis. Apparently, though not unequivocally tested, there are temporal waves of brown adipogenesis from different APCs. *Pdgfra*-expressing APCs, including *Dpp4^+^* fascial cells, form interscapular BAT starting around embryonic day 14.5 in mice^20,21^. BAT expands rapidly after birth^22^, and *Pdgfrb*^+^ and *Tbx18*^+^ pericytes are the early postnatal APCs for BAT growth^23^. While the adipogenic potential of *Pdgfra*^+^ and *Pdgfrb*^+^ APCs can be unlocked by cold in adult mice^24–26^, their contribution to BAT homeostasis in animals housed at room temperature (RT) is rather limited^26–30^. In addition, a group of smooth muscle-like cells expressing *Trpv1* and *Myh11* is also an adipogenic source of interscapular and perivascular BAT^21,31^. And a subset of UCP1^+^ brown adipocytes can proliferate and contribute to BAT expansion^32,33^. However, the temporal relationship and division of labor between these heterogeneous groups of APCs are not known. Importantly, it remains undetermined if specific APCs exist during aging, in order to replenish dead brown adipocytes and support tissue turnover.

Growing evidence starts to reveal the metabolic heterogeneity of brown adipocytes. Compared to classic *Ucp1*-highly expressing brown adipocytes, a group of low-thermogenic brown adipocytes co-exist in BAT with larger lipid droplets and lower mitochondrial content^34^. Low-thermogenic brown adipocytes become abundant in thermoneutrality and aging^34,35^. On the other hand, cold exposure actively alters the composition of brown adipocyte subtypes^36^, and a group of lipogenic brown adipocytes may mediate thermogenic memory^37^. Furthermore, a subpopulation of *Cyp2e1*+ adipocytes, though rare in BAT, can robustly regulate the thermogenic capacity of classic brown adipocytes^38^. Despite these rapid advances in our understanding of the metabolic plasticity of BAT, it is unknown if such heterogeneity within mature brown adipocytes is established and influenced by progenitor diversity.

In this study, taking advantage of scRNA-seq, lineage tracing, and genetic cell ablation in mice, we uncover an age-dependent switch from embryonic *Pdgfra*^+^ APCs to adult *Myl1*^+^ APCs in maintaining BAT homeostasis. Brown adipocytes derived from *Myl1*^+^ APCs display unique molecular signatures, constitute BAT heterogeneity, and are essential for preserving BAT mass and metabolic fitness in midlife. Our study establishes the *Myl1*^+^ lineage as a preferable target for future strategies to recruit or engineer BAT.

## Results

### Minimal BAT adipogenesis from *Pdgfra*^+^ APCs in adult mice at room temperature.

**Figure 1:**
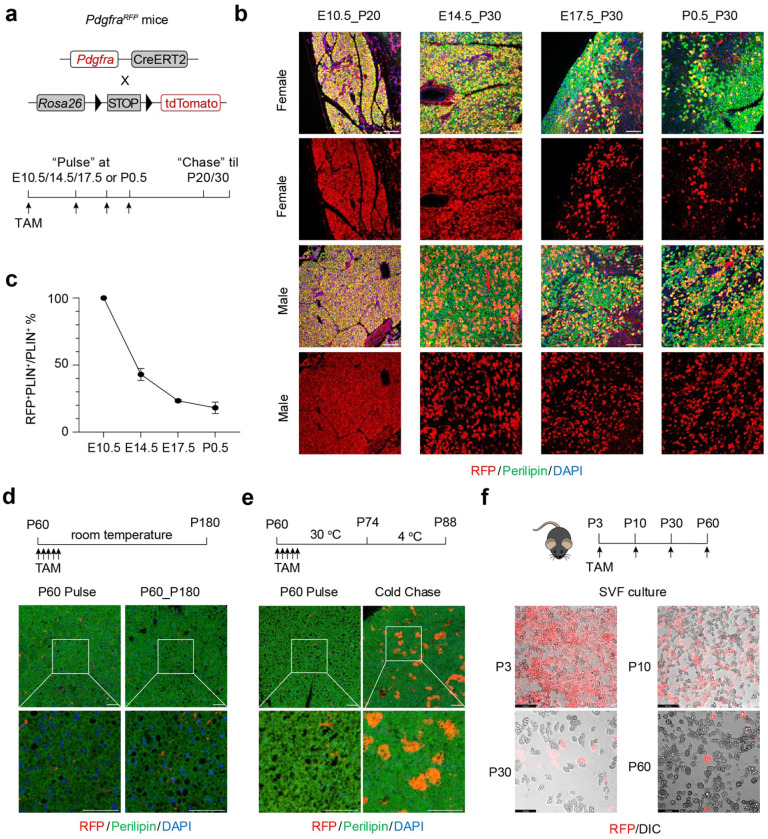
*Pdgfra*^+^ APCs progressively lose their ability to give rise to brown adipocytes. **a**, *Pdgfra*^*RFP*^ (*Pdgfra*^*CreERT2*^*Rosa26*^*RFP*^) mice were administered once with TAM at indicated time until P20 or P30. **b**, Representative immunofluorescence staining of iBAT sections from *Pdgfra*^*RFP*^ mice chased at different time points. **c**, Quantification of the percentage of RFP^+^ brown adipocytes in **b**. **d**, Representative immunofluorescence staining of iBAT sections from *Pdgfra*^*RFP*^ mice following pulse labeling and room-temperature chase from P60 to P180. **e**, Representative immunofluorescence staining of iBAT sections from *Pdgfra*^*RFP*^ mice after pulse labeling and 2-week cold chase. **f**, Representative DIC/RFP images of differentiated SVF cells isolated from BAT depots from *Pdgfra*^*RFP*^ mice pulsed at different ages. Red, RFP; green, Perilipin; blue, DAPI. Scale bar, 50 μm.

The temporal contribution of *Pdgfra*^+^ APCs to BAT early development and adult homeostasis has not been explicitly investigated. Using the *Pdgfra^RFP^*(*Pdgfra^CreERT2^; Rosa26^tdTomato^*) fate mapping mice, we recently showed that *Pdgfra*^+^ APCs are essential to postnatal adipose tissue development but not to its adult formation^28^. Here, we performed temporal “pulse-chase” lineage tracing of BAT using the *Pdgfra^RFP^*mice housed at room temperature (RT, Fig. 1a). Brown adipocytes derived from *Pdgfra*^+^ APCs, as assessed by RFP/UCP1/Perilipin co-staining, were predominant in interscapular BAT (iBAT) at postnatal day 20 (P20) when pulsed at the embryonic day 10.5 (E10.5) (Fig. 1b). Notably, the contribution of *Pdgfra*^+^ APCs to BAT progressively declined if they were traced at later stages, including E14.5, E17.5, and postnatal day 0.5 (P0.5), in both females and males (Fig. 1b, c). Strikingly, the P60–P180 chase in adult mice produced minimal RFP^+^ adipocyte labeling in BAT (Fig. 1d), suggesting that *Pdgfra*^+^ APCs make little to no contribution to brown adipocytes in adulthood.

The low tracing rate of *Pdgfra*^+^ APCs in adult mice was unlikely due to the lack of adipogenesis, since multiple reports have demonstrated active BAT turnover during adulthood even at RT^23,34^. To confirm the fidelity of the *Pdgfra^RFP^* line, we pulsed P60 mice and housed them at thermoneutrality (TN, 30°C) for 2 weeks, followed by another 2 weeks of cold challenge at 4°C (Fig. 1e). As expected, many iBAT adipocytes were labelled RFP (Fig. 1e), indicative of active adipogenesis from *Pdgfra*^+^ APCs induced by cold.

To directly test the adipogenic capacity of *Pdgfra*^+^ APCs, we labeled them with tamoxifen in RT -housed *Pdgfra^RFP^* mice at P3, P10, P30, and P60, isolated and differentiated stromal vascular fraction (SVF) cells *in vitro* (Fig. 1f). By assessing RFP overlapped with lipid-containing adipocytes, we confirmed that *Pdgfra*^+^ cells exhibited minimal brown adipogenic potential in adulthood when compared to the early postnatal period (Fig. 1f). We conclude from these results that *Pdgfra*^+^ APCs are crucial for early development but do not actively differentiate in adult adipose tissue. Recruitment and activation of *Pdgfra*^+^ APCs require adipogenic stimuli such as high-fat feeding (in WAT) and increased thermogenic demand (in brown and beige adipose tissue) ^25,39–41^.

### *Pdgfra*^+^ APCs contribute little to adult BAT homeostasis.

**Figure 2:**
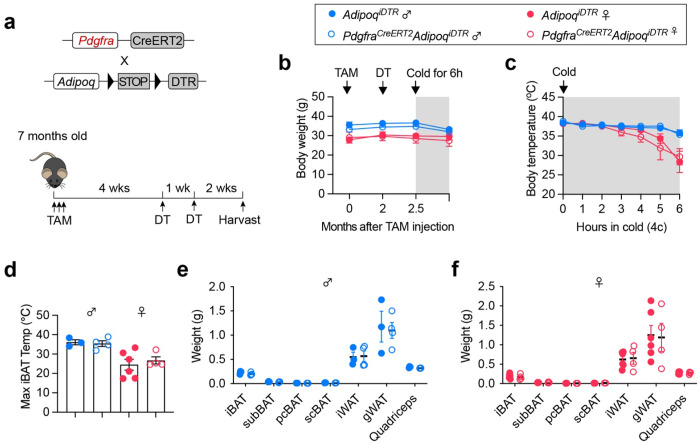
*Pdgfra*+ APCs are dispensable for BAT maintenance in adult mice **a**, Generation of inducible “deleter” mice specifically for *Pdgfra*^+^ APC-derived adipocytes. **b**, Body weight of 7-month-old female (n = 4-6) and male (n = 3-4) *Pdgfra*^*CreERT2*^ *Adipoq*^*iDTR*^ mice after TAM administration, DT injection, and 6-hour cold exposure. **c**, Body temperature of control and *Pdgfra*^*CreERT2*^ *Adipoq*^*iDTR*^ mice during cold exposure with food deprivation. **d**, Maximal iBAT temperature after 6 hours of cold challenge. **e,f**, Tissue weight of male (e) and female (f) *Pdgfra*^*CreERT2*^ *Adipoq*^*iDTR*^ mice at the end of experiment. Data are mean ± SEM.

To determine the function of *Pdgfra*^+^ APCs in adult BAT, we generated an inducible adipocyte “deleter” mice (*Adipoq^iDTR^*) that express diphtheria toxin receptor (DTR) under the control of the *Adipoq* promoter in a Cre-dependent manner and crossed them to the *Pdgfra^CreERT2^* (Fig. 2a). Tamoxifen (TAM) injection to 7-month-old *Pdgfra^CreERT2^ Adipoq^iDTR^* mice induced DTR expression in newly formed adipocytes derived from *Pdgfra*^+^ APCs over a 4-week chase period, followed by two doses of diphtheria toxin (DT) to ablate these adipocytes. No differences in body weight between *Adipoq^iDTR^* (control) and *Pdgfra^CreERT2^ Adipoq^iDTR^* mice were observed in either males or females (Fig. 2b). *Pdgfra^CreERT2^ Adipoq^iDTR^* mice were able to maintain their body temperature (Fig. 2c) and iBAT temperature (Fig. 2d) during acute cold challenge. Ablation of *Pdgfra*^+^ APC-derived adipocytes, if any, did not change tissue weight of various BAT and WAT depots, including iBAT, subscapular (sub) BAT, posterior cervical (pc) BAT, supraclavicular (sc) BAT, inguinal (i) WAT, and gonadal (g) WAT (Fig. 2e, f). Together, these data suggest that *Pdgfra*^+^ APCs play a minimal role in maintaining BAT homeostasis and function at the steady state.

### Identification of *Myl1*-expressing APCs.

**Figure 3:**
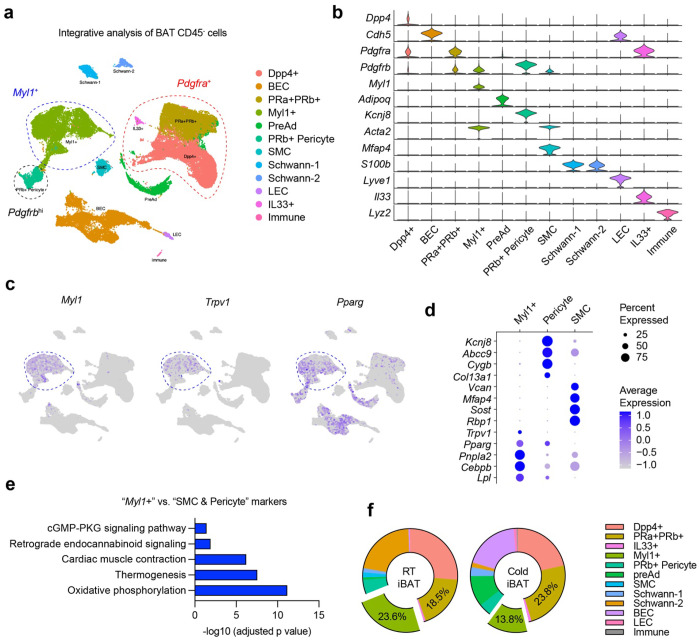
Identification of *Myl1*^+^ APCs from scRNA-seq data **a**, UMAP plot of CD45- SVF cells identifying major cell populations in BAT. *Myl1*^+^, *Pdgfra*^+^, and *Pdgfrb*^*hi*^ cells are circled. **b**, Violin plot of marker gene expression across cell populations. **c**, UMAP plots showing *Myl1*, *Trpv1*, and *Pparg* gene expression. **d**, Dot plot comparing the expression of lipid metabolism genes, pericyte markers, and SMC markers between *Myl1*^+^ APCs, pericytes, and SMCs. **e**, Pathway enrichment of genes highly expressed in *Myl1*^+^ APCs compared to pericytes and SMCs. **f**, Frequencies of individual cell populations in BAT from mice housed at RT or cold-exposed.

To identify alternative sources of brown adipogenesis within the stromal compartment, we performed an integrated analysis of scRNA-seq data from BAT SVF cells from three groups (Granneman^24^, Tseng^31^, and Seale^21^). Clustering of CD45 negative cells identified all major stromal subtypes (Fig. 3a, b, and Fig. S1a, b), including *Pdgfra*^+^/*Dpp4*^+^ interstitial progenitors^42^, *Pdgfra*^+^/*Pdgfrb*^+^ APCs^23^, *Il33*^+^/*Pdgfra*^+^ stromal cells^43^, *Pdgfrb*^hi^ pericytes^44^, *Acta2*^+^/*Myh11*^+^ smooth muscle cells (SMCs), *Adipoq*^+^/*Ucp1*^+^ brown (pre)adipocytes, blood endothelial cells (BECs), lymphatic endothelial cells (LECs), and Schwann cells. Notably, within this landscape, we identified a discrete population of *Acta2*^+^/*Myh11*^+^/*Pdgfra*^−^/*Pdgfrb*^lo^ cells uniquely labelled by *Myl1*, which encodes a striated muscle myosin light chain (Fig. 3b, c). These *Myl1*^+^ cells did not express canonical markers for SMCs (e.g., *Kcnj8* and *Abcc9*) or pericytes (e.g., *Vcan* and *Mfap4*); instead, they expressed vascular smooth muscle-derived progenitor marker *Trpv1*^31^, together with adipogenic regulators (*Pparg* and *Cebpb*) and lipid metabolism genes (*Lpl* and *Pnpla2*) (Fig. 3c, d, and Table S1). Compared to *Pdgfrb*-expressing SMCs and pericytes, pathway analysis indicated that genes enriched in the *Myl1*^+^ cluster (fold change > 2; >50% expressing) were associated with oxidative phosphorylation, thermogenesis, and muscle contractile programs (Fig. 3e). Moreover, *Myl1*^+^ cells were the dominant stromal population that express niche cytokines, including *Pdgfa, Notch3*, and *Jag1* (Fig. S1c), ligands known to restrain brown adipogenesis ^26,28,45^, suggesting potential regulatory crosstalk between *Myl1*^+^ cells and classical *Pdgfra*^+^/*Pdgfrb*^+^ APCs.

To assess depot specificity, we interrogated an integrated scRNA and single-nucleus(sn) RNA-seq WAT atlas containing ~300,000 cells from visceral and subcutaneous WAT^46^. We found no expression or enrichment of *Myl1* in any stromal or mature adipocyte populations (Fig. S2), suggesting that *Myl1*^+^ cells are uniquely enriched in BAT. Finally, we also quantified stromal populations found in iBAT scRNA-seq from mice housed at RT or cold for 2-7 days. As previously reported, cold exposure increased *Pdgfra*^+^/*Pdgfrb*^+^ APC proportions; however, a higher percentage of *Myl1*^+^ cells was observed at RT than cold housing (Fig. 3f), suggesting that *Myl1*^+^ progenitors might be more active at the steady state when mice are adapted to the mild cold stress at RT.

### Age-dependent adipogenesis from *Myl1*^+^ APCs

**Figure 4:**
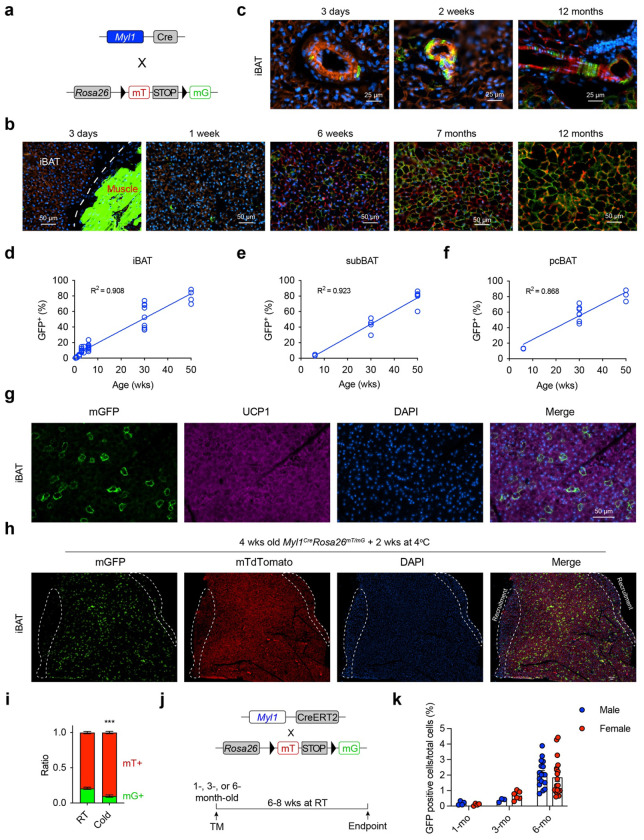
Fate mapping of *Myl1*^+^ APCs in BAT. **a**, Generation of the *Myl1*^*Cre*^ *Rosa26*^*mT/mG*^ reporter mice. **b**, Representative images showing perivascular mGFP^+^ cells in iBAT from mice at the indicated ages. **c**, Representative immunofluorescent images of iBAT showing increasing numbers of mGFP^+^ brown adipocytes as mice age. Note the extensive labeling of peri-BAT skeletal muscle cells in P3 mice. **d-f**, Quantification of mGFP^+^ brown adipocyte percentage in iBAT (d), subBAT (e), and pcBAT (f) as a function of age. **g**, Co-staining of UCP1 and mGFP^+^ on the iBAT section from 4-week-old *Myl1*^*Cre*^ *Rosa26*^*mT/mG*^ mice. **h-i**, 4-week-old *Myl1*^*Cre*^ *Rosa26*^*mT/mG*^ mice were subjected to 2 weeks of housing at 4°C, the whole lobe of iBAT was imaged for mGFP, mTdTomato, and DAPI (h). Regions of BAT recruitment at the edge, with stronger DAPI staining, were circled in dotted lines. The percentage of mGFP^+^ and mTdTomato^+^ adipocytes in the whole BAT was quantified in (i). **j**, Generation of the inducible *Myl1*^*CreERT2*^ *Rosa26*^*mT/mG*^ reporter mice and timelines for the pulse-chase lineage tracing. **k**, Quantification of mGFP^+^ adipocytes in iBAT from male and female *Myl1*^*CreERT2*^ *Rosa26*^*mT/mG*^ mice pulsed at 1-, 3-, or 6-month of age and chased for 6-8 weeks. Data are mean ± SEM. P values were calculated by a two-tailed unpaired Student’s *t*-test. ***P < 0.001.

To examine if *Myl1*^+^ cells could function as brown APCs, we first performed cell marking by crossing a constitutive *Myl1^Cre^* to the *Rosa26^mT/mG^* reporter mice (Fig. 4a). As a myocyte-specific line, *Myl1^Cre^* consistently labeled skeletal muscle cells as expected (Fig. 4b). Notably, a subpopulation of mGFP+ perivascular cells could be observed in iBAT from newborn to middle-aged mice (Fig. 4c). No mGFP+ brown adipocytes were detected at postnatal day 3, but scattered lineage-marked adipocytes started to be seen in 1-2 weeks old pups (Fig. 4b). The percent of *Myl1*-labeled adipocytes increased to ~15% at 6 weeks, ~50% at 7 months, and ~80% at 12 months in iBAT (Fig. 4b, d). A similar pace and percentage of *Myl1*^+^ marked brown adipocytes could also be observed in the subscapular (sub) and posterior cervical (pc) BAT depots (Fig. 4e, f). To confirm that mGFP-labeled adipocytes were bona fide brown adipocytes, we co-stained for UCP1 and found that mGFP^+^ and unlabeled adipocytes expressed comparable levels of UCP1 protein (Fig. 4g), validating their brown identity. We next asked whether age-related changes in Myl1 expression might account for the increased lineage labeling. Compared with the SVF fraction, mature brown adipocytes expressed little MYL1 protein, and this low level did not increase with aging (Fig. S3a). *Myl1* transcription in SVF cells was likewise comparable between young and old mice (Fig. S3b). Thus, the age-dependent rise in labeling reflects cumulative differentiation from *Myl1*^+^ progenitors rather than increased *Myl1* expression in mature adipocytes. Together, these data indicate that *Myl1^+^* APCs contribute minimally to neonatal BAT but become the dominant progenitor pool in adulthood.

To assess whether *Myl1^+^* APCs contribute to cold-induced adipogenesis, we challenged 4-week-old *Myl1^Cre^; Rosa26^mT/mG^* mice with 2 weeks of cold exposure to induce *de novo* brown adipogenesis. As previously reported^25^, newly formed brown adipocytes were on the dorsal edge of the iBAT depot and stained strongly with DAPI (Fig. 4h). Notably, these cold-recruited regions were largely devoid of mGFP^+^ adipocytes (Fig. 4h), and cold exposure modestly reduced the overall proportion of *Myl1*-derived adipocytes (Fig. 4i). This aligns with scRNA-seq data showing a relative depletion of *Myl1^+^* APCs in cold-stimulated BAT (Fig. 3f) and supports the existing dogma that *Pdgfra*^+^ and *Pdgfrb*^+^ progenitors are the primary source of cold-induced brown adipogenesis.

To perform temporal “pulse-chase” lineage tracing, we bred the *Rosa26^mT/mG^* reporter to an inducible *Myl1^CreERT^* line (Fig. 4j). *Myl1^CreERT2^; Rosa26^mT/mG^* mice were pulsed with tamoxifen at 1, 3, and 6 months of age and chased for 6-8 weeks at RT. In both males and females, we observed an age-dependent increase in the differentiation of *Myl1*^+^ APCs into mature adipocytes (Fig. 4k and Fig. S3c). Together, these findings demonstrate that *Myl1^+^* cells are bona fide APCs that contribute minimally during early life but increase their contribution to brown adipogenesis under homeostatic conditions in adulthood.

### *Myl1*^+^ APCs are essential sources of adult brown adipocytes

**Figure 5:**
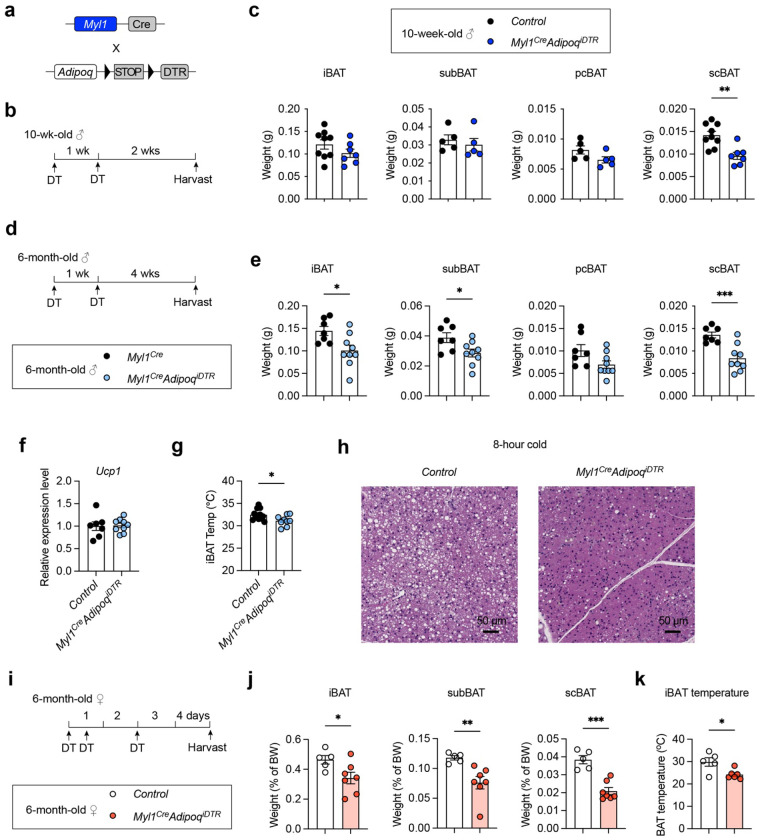
*Myl1*^+^ APCs maintain BAT mass and function in adult mice. **a**, Generation of inducible “deleter” mice specifically for *Myl1*^+^ APC-derived adipocytes. **b-c**, Two doses of DT were administered to 10-week-old male *Myl1*^*Cre*^ *Adipoq*^*iDTR*^ mice (b, n = 5), and the weights of the indicated BAT depots were measured after 2 weeks (c). **d-h**, Two doses of DT were administered to 6-month-old male *Myl1*^*Cre*^ *Adipoq*^*iDTR*^ mice (d, n = 7-9). BAT weight was measured after 4 weeks (e). Relative *Ucp1* gene expression in iBAT was determined by RT-qPCR (f). Average iBAT temperature was measured after 6 hours of cold exposure (g, n = 9-12). Representative H&E staining of iBAT from mice challenged with cold for 6 hours. **i-k**, DT was administered to 6-month-old female *Myl1*^*Cre*^ *Adipoq*^*iDTR*^ mice (n = 5-7) for cell ablation (i). BAT weight (j) and iBAT temperature after cold challenge (k) were measured. Data are mean ± SEM. P values were calculated by a two-tailed unpaired Student’s *t*-test. *P < 0.05, **P < 0.01 and ***P < 0.001.

To evaluate the functional relevance of *Myl1*^+^ APCs to BAT, we utilized the inducible adipocyte “deleter” mice (*Adipoq^iDTR^*) and crossed them to *Myl1*^Cre^ (Fig. 5a). We first ablated *Myl1*^+^ APC-derived adipocytes in young 10-week-old males with two doses of DT (Fig. 5b). No differences in body weight, WAT, or muscle mass (Fig. S4a) were noticed, suggesting no overall adversity or leakage of the model. Consistently with the low *Myl1*^+^ labeling at this age, BAT depots in interscapular, subscapular and posterior cervical regions were not evidently perturbed by DT/DTR-mediated ablation (Fig. 5c). Interestingly, a modest reduction of supraclavicular BAT (scBAT) in young *Myl1^Cre^ Adipoq^iDTR^* mice was observed, suggesting that *Myl1*^+^ APCs may participate in the early establishment of this anatomically distinct and clinically relevant depot, a possibility warranting further investigation.

We next performed adipocyte deletion in 6-month-old *Myl1^Cre^ Adipoq^iDTR^* male mice (Fig. 5d). Administration of DT did not alter the body weight, WAT, or skeletal muscle (Fig. S4b). Instead, a 30-50% reduction of BAT mass was evident in all depots examined (Fig. 5e), matching the lineage marking percentage at this age (Fig. 4). Despite the BAT paucity in these animals, relative *Ucp1* gene expression in the remaining tissue was compared to controls (Fig. 5f). Reduced iBAT mass in DT-injected *Myl1^Cre^ Adipoq^iDTR^* mice resulted in a reduction of iBAT temperature during cold challenge (Fig. 5g). Histological examination of iBAT from *Myl1^Cre^ Adipoq^iDTR^* mice revealed a profound depletion of intracellular lipids after cold exposure (Fig. 5h), suggesting increased fat utilization to compensate for the BAT mass loss. It also indicates a different scenario in which *Myl1*^+^ APCs give rise to a distinct subset of brown adipocytes that disproportionately rely on lipid metabolism to support thermogenesis in adulthood.

In addition, we also deleted *Myl1*-lineage adipocytes in 6-month-old females (Fig. 5i), using an acute DT-mediated ablation protocol^47^. We observed a significant reduction of iBAT, subBAT, and scBAT weight (Fig. 5j), without negative impact on body weight change, WAT, or muscle weight (Fig. S4c). As a result, iBAT from female *Myl1^Cre^ Adipoq^iDTR^* mice displayed lower temperature after 6 hours of cold challenge (Fig. 5k). These results demonstrate that *Myl1*^+^ APCs are important sources for adult BAT maintenance.

### *Myl1+* APC-derived brown adipocytespossess a unique molecular signature

**Figure 6:**
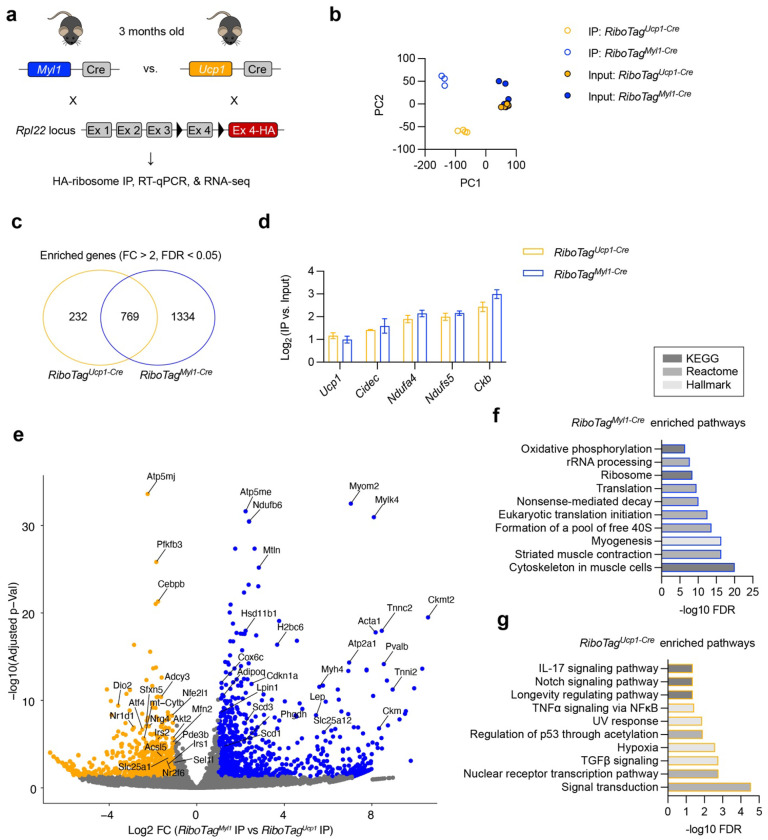
Ribosomal profiling of *Myl1*^+^ APC-derived brown adipocytes **a**, RiboTag mouse lines and experimental procedures for ribosomal profiling. **b**, Principal component analysis (PCA) plot for gene expression of input and ribosomal IP samples from *RiboTag*^*Myl1-Cre*^ and *RiboTag*^*Ucp1-Cre*^ mice. **c**, Venn diagram showing numbers of significantly enriched genes in *RiboTag*^*Myl1-Cre*^ and *RiboTag*^*Ucp1-Cre*^ BAT. **d**, Enrichment of selected thermogenic and mitochondrial genes in ribosomal IP compared to total input. **e**, Volcano plot to visualize differentially transcribing genes between *RiboTag*^*Myl1-Cre*^ and *RiboTag*^*Ucp1-Cre*^ ribosomes. **f, g**, Pathway analysis (KEGG, Reactome, and Hallmark pathways) of *RiboTag*^*Myl1-Cre*^ ribosome-enriched genes (f) and *RiboTag*^*Ucp1-Cre*^ ribosome-enriched genes (g). Data are mean ± SEM. P values were calculated by a two-tailed unpaired Student’s *t*-test.

To determine if brown adipocytes derived from *Myl1*^+^ APCs exhibit distinct molecular features, we performed ribosomal profiling to analyze cell-type-specific mRNA translation^48^. The “*RiboTag*” mice carrying a Cre-dependent HA-tagged ribosomal protein L22 (*Rpl22*) locus were crossed to *Myl1^Cre^*, allowing HA immunoprecipitation (IP) to isolate actively translating polyribosomes from *Myl1*^+^ APCs and their descendants (Fig. 6a). For comparison, *Ucp1^Cre^* mediated *RiboTag* mice were generated to profile active mRNA translation in all brown adipocytes. Total iBAT samples from 3-month-old adult animals that had an adequate number of (~30%) *Myl1*^+^ APC-derived adipocytes were collected for analysis. RT-qPCR showed a similar enrichment of *Ucp1, Dio2*, and *Fabp4* genes in *RiboTag^Myl1-Cre^*and *RiboTag^Ucp1-Cre^*ribosomes when normalized to input mRNA (Fig. S5a), while stromal genes (e.g., *Fstl1*^49^) were depleted, confirming experimental validity. We then performed RNA-seq, and principal component analysis (PCA) showed a distant separation between ribosomal IP samples from *RiboTag^Myl1-Cre^*and *RiboTag^Ucp1-Cre^*BAT (Fig. 6b). As expected, 4-way analysis revealed a significant overlap between *RiboTag^Myl1-Cre^*and *RiboTag^Ucp1-Cre^*ribosomal genes (Fig. 6c and Table S2), many of which were adipocyte, mitochondrial, and thermogenic markers, such as *Ucp1, Adipoq, Cidec, Fabp4, Ndufa4, Ndufa6*, and *Ckb* (Fig. 6d and Fig. S5b). On the other hand, markers for the SVF compartment, including APCs, endothelial cells, and immune cells, were similarly depleted between *RiboTag^Myl1-Cre^* and *RiboTag^Ucp1-Cre^* ribosomes (Fig. S5b-d).

We then analyzed differential genes between *RiboTag^Myl1-Cre^* and *RiboTag^Ucp1-Cre^*IP samples (Fig. 6e, Fig. S5e, and Table S3). Pathway analysis revealed that *RiboTag^Myl1-Cre^* ribosomes enriched genes involved in myogenesis and muscle contraction (likely due to the targeting of intra-BAT myocytes by *Myl1^cre^*), RNA/ribosome processing and translation, and oxidative phosphorylation (Oxphos) (Fig. 6f). Significantly upregulated genes from *RiboTag^Myl1-Cre^* ribosomes encodes many Oxphos complex genes, such as *Ndufb6, Ndufa12, Ndufv3, Uqcr11, Cox5b, Cox6c*, and *Cox7b* (Fig. 6e and Table S3). Conversely, signaling transduction pathways relevant to thermogenesis, such as TGFβ, nuclear receptor, Notch, and TNFα signaling, were overrepresented in *RiboTag^Ucp1-Cre^* ribosomal genes (Fig. 6g). Notably, genes related to insulin signaling (e.g., *Irs1, Irs2*, and *Akt2*) and glycolysis (e.g., *Pfkfb3* and *Pdk1*) were less actively translated by *RiboTag^Myl1-Cre^* ribosomes (Fig. 6e and Table S3). Together, these data reveal that *Myl1*^+^ APC-derived brown adipocytes adopt a distinct translational and metabolic identity characterized by enhanced oxidative phosphorylation and reduced glycolytic and insulin signaling programs.

### *Myl1*^+^ APCs may give rise to Oxphos-high brown adipocytes

**Figure 7:**
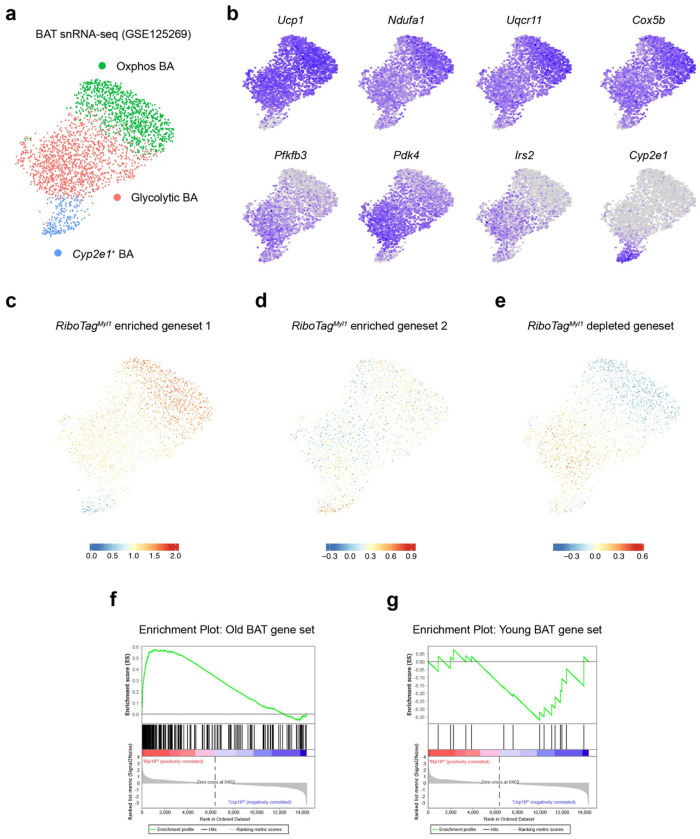
BAs derived from *Myl1*^+^ APCs possess gene signatures for Oxphos BAs and aging **a**, UMAP plot showing three main brown adipocyte (BA) populations identified by snRNA-seq. **b**, Gene expression visualization across cells with UMAP plots. **c-e**, Combined expression (module scores) of top enriched *RiboTag*^*Myl1-Cre*^ gene set-1 (c), −2 (d), and *RiboTag*^*Ucp1-Cre*^ gene set (e). **f, g**, Enrichment plots for gene sets higher in old (f) and young (g) BAT for the GSEA (Gene Set Enrichment Analysis) of differentially expressed genes between *RiboTag^Myl1-Cre^* and *RiboTag^Ucp1-Cre^* ribosomes.

To determine how *Myl1*^+^ APC-derived adipocytes relate to known brown adipocyte subtypes, we reanalyzed a previously published snRNA-seq dataset of BAT^34^. After excluding non-adipocytes (Fig. S6a, b), three major subsets of brown adipocytes emerged at the UMAP space (Fig. 7a and Table S4): The first comprised *Ucp1-high* “Oxphos” adipocytes, which were enriched for mitochondrial respiratory chain genes (e.g., *Ndufa1, Uqcr11, Cox5b*). The second consisted of “glycolytic” adipocytes expressing glycolytic enzyme genes (*Pfkfb3, Pdk4, Gk*) and insulin signaling components (*Irs2, Foxo1*) (Fig. 7b). The third group, marked uniquely by *Cyp2e1*, corresponded to the recently described regulatory adipocyte population^38^.

We next compared these transcriptional subtypes with the *RiboTag^Myl1-Cre^*ribosome-enriched genes and listed the top ones that were expressed by mature adipocytes (Table S5). These genes mapped predominantly to Oxphos or *Cyp2e1*^+^ adipocytes (Fig. 7c, d). On the other hand, *RiboTag^Myl1-Cre^*ribosome-depleted genes were expressed by glycolytic and *Cyp2e1*^+^ adipocytes (Fig. 7e and Table S6). These patterns raise the possibility that Oxphos and glycolytic brown adipocytes arise preferentially from *Myl1*+ and non-*Myl1*^+^ APCs, respectively, whereas the regulatory *Cyp2e1*^+^ subtype may have mixed lineage origins.

To assess whether *Myl1*-derived adipocytes become more prominent with age, we analyzed the transcriptomes of BAT from young (2–4 months) and old (24 months) mice in two independent datasets^50,51^. Differentially expressed gene sets were used for GESA analysis (Table S7). We found that gene sets upregulated with aging were significantly enriched for *RiboTag^Myl1-Cre^*ribosomal genes (Fig. 7f), whereas aging-downregulated gene sets were enriched for *RiboTag^Ucp1-Cre^*ribosomal genes (Fig. 7g). These findings support the conclusion that *Myl1*^+^ APC-derived brown adipocytes increasingly dominate the adipocyte landscape during aging.

### *Myl1*^+^ APC adipogenesis is required to maintain BAT integrity and glucose control in aging

**Figure 8:**
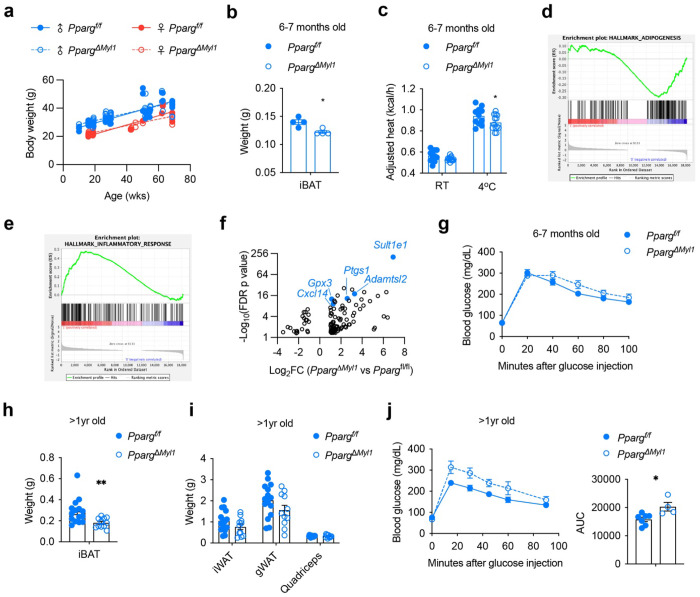
*Myl1*^+^ APC adipogenesis controls BAT mass and glucose metabolism in old mice **a**, Body weight of *Pparg*^*f/f*^ and *Pparg*^*ΔMyl1*^ mice in both sexes. **b**, iBAT weight of 6-7 months old *Pparg*^*f/f*^ and *Pparg*^*ΔMyl1*^ male mice (n = 4). **c**, *Pparg*^*f/f*^ and *Pparg*^*ΔMyl1*^ male mice were subjected to metabolic cage analysis at RT, followed by a 4°C cold challenge for 1 day. Daily heat production using ANCOVA analysis (adjusted to body weight) was calculated (n = 11-13). **d-f**, RNA-seq of iBAT was performed. Enrichment plots of adipogenesis (d) and inflammatory response (e) gene sets that are upregulated in *Pparg*^*f/f*^ and *Pparg*^*ΔMyl1*^ BAT, respectively. (f) Volcano plot of differentially expressed genes, with *Pdgfra*^+^ APC marker genes highlighted. **g**, Glucose tolerance test of 6-7 months old *Pparg*^*f/f*^ and *Pparg*^*ΔMyl1*^ male mice (n = 8-11). **h, i**, Weights of iBAT (h), WAT, and quadriceps (i) of *Pparg*^*f/f*^ and *Pparg*^*ΔMyl1*^ male mice that were 12-21 months old (n = 11-13). **j**, Glucose tolerance test of 18-month-old *Pparg*^*f/f*^ and *Pparg*^*ΔMyl1*^ male mice (n = 4-7). Area under curve (AUC) was calculated and shown to the right. Data are mean ± SEM. P values were calculated by a two-tailed unpaired Student’s *t*-test. *P < 0.05 and **P < 0.01.

To assess the physiological importance of the differentiation from *Myl1*^+^ APCs into adipocytes, we knocked out the master adipogenic transcription factor PPARγ driven by the *Myl1^Cre^*. No change in body weight was observed in both female and male *Pparg^ΔMyl1^*, compared to *Pparg^f/f^* controls (Fig. 8a). By 6 to 7 months of age, a modest but significant reduction in iBAT weight was observed in male *Pparg^ΔMyl1^* (Fig. 8b). This was associated with reduced heat production when challenging *Pparg^ΔMyl1^* males with cold (Fig. 8c). RNA-sequencing followed with GESA analysis revealed that PPARγ deficiency was negatively correlated with adipogenesis, while positively linked to inflammation (Fig. 8d, e, and Table S8). Several upregulated transcripts were markers of *Pdgfra*^+^ APCs, including *Gpx3, Cxcl12, Ptgs1, Sult1e1*, and *Adamtsl2* (Fig. 8f), suggesting that compensatory recruitment of *Pdgfra*^+^ APCs may occur when *Myl1*^+^ APCs differentiation is blocked. When subjected to a glucose tolerance test, *Pparg^ΔMyl1^* animals showed a subtle increase in blood glucose levels (Fig. 8g).

We next examined the impact of long-term loss of *Myl1*-lineage adipogenesis. After more than 1 year of aging, a consistent reduction in iBAT mass was found in both male (Fig. 8h) and female *Pparg^ΔMyl1^* mice (Fig. S7a). As expected, WAT and skeletal muscle weight remained unaffected in old males (Fig. 8i) and females (Fig. S7b), suggesting that *Myl1*^+^ APC was not an essential source for white adipogenesis and PPARγ was dispensable for myocytes. Notably, aged *Pparg^ΔMyl1^* males displayed significantly impaired glucose tolerance (Fig. 8j). Collectively, these findings demonstrate that adipogenesis from *Myl1*^+^ APCs is essential for maintaining BAT mass and supporting metabolic health during aging, highlighting their importance as a protective progenitor pool in later life.

## Discussion

BAT undergoes continuous remodeling across the lifespan, yet the progenitor sources sustaining this turnover have remained largely undefined. Here we identify an age-dependent progenitor-switching mechanism in which embryonic and early postnatal *Pdgfra*^+^ APCs establish BAT, whereas a distinct population of *Myl1*^+^ APCs progressively becomes the dominant source of brown adipocytes in adulthood. This transition provides a mechanistic framework that explains why early APCs contribute minimally to homeostatic brown adipogenesis in adulthood but are rather primarily recruited in response to strong thermogenic stimuli.

Waves of cellular differentiation from distinct types of stem and progenitor cells across the lifespan are common phenomena. In many systems, fetal programs and progenitors can be reactivated following injury and stress^52^. For instance, *Lgr5*^+^ intestinal stem cells are replaced by fetal-like revival stem cells upon helminth infection, which is crucial for type 2 immune regulation^53,54^. Similar principles likely apply to adipose tissues. We propose that acute cold-induced adipogenic differentiation from early *Pdgfra*^+^ and/or *Pdgfrb*^+^ APCs represents a stress-activated fetal programming in BAT. While in mice housed at thermoneutrality or adapted to the mild cold at RT, adipogenesis from *Myl1*^+^ APCs supports BAT homeostasis and turnover. Such temporal transitions (developmental vs. adult) and context-dependent utilization (homeostasis vs. obesity and aging) of APCs are also increasingly recognized in WAT^55,56^.

*Myl1*^+^ APCs form a transcriptionally distinct, BAT-restricted stromal population enriched for Oxphos genes, niche ligands, and adipogenic regulators, while lacking canonical markers of SMCs. Consistent with the findings from scRNA-seq integration, lineage tracing experiments have repeatedly shown that SMCs labelled by *Myh11* or *Acat2* do not give rise to iBAT adipocytes^21,57,58^. Instead, a distinct population of SMC-like cells marked by *Trpv1*^31^ and *Myl1* (this study) are adipogenic. Our current study demonstrates for the first time the age-dependent recruitment of *Myl1*^+^ APCs. More importantly, progenitor origin underlies metabolic heterogeneity within BAT: *Myl1*^+^ APCs generate OxPhos-high adipocytes, whereas non-*Myl1* progenitors contribute more glycolytic populations, with regulatory *Cyp2e1*^+^ adipocytes likely reflecting a mixed lineage. Future experiments incorporating fate mapping with single-cell metabolic and functional profiling are required to determine the route of BAT heterogeneity.

*Myl1^+^* APCs are essential for maintaining BAT mass, preserving thermal homeostasis, and sustaining glucose control during aging. Conditional deletion of *Pparg* in *Myl1^+^* APCs abolished their differentiation into brown adipocytes, resulting in marked BAT loss, cold intolerance, and impaired glucose metabolism. These findings indicate that *Myl1^+^* APCs are not only recruited in adulthood but also function as obligately PPARγ-dependent progenitors whose adipogenic output is particularly critical for BAT integrity during aging.

Despite these insights, several mechanistic questions remain. The developmental origin of *Myl1*^+^ APCs is still unresolved. They may represent a fate-restricted derivative of embryonic *Pdgfra*^+^ stromal progenitors that undergo lineage maturation, or an independent mesenchymal lineage emerging from muscle-associated or vascular smooth muscle–derived progenitors. The stability of *Myl1* expression across age and its absence in neonatal APCs argue against simple activation of the *Pdgfra*^+^ lineage. Resolving whether this lineage transition is developmentally programmed or environmentally induced will require a temporally resolved dual-recombinase that provides resolution beyond what classical Cre-based lineage tracing can achieve.

Our data also raises the question of what signals activate *Myl1*^+^ APCs. Their enrichment at room temperature and depletion during acute cold exposure suggest that mild, chronic thermogenic demand, not β-adrenergic stimulation, governs their recruitment. *Myl1*^+^ APCs express PDGF, Notch, and TGFβ ligands, which are capable of modulating progenitor competence, raising the possibility of autocrine or paracrine regulation. The mitochondrial metabolic state may further influence APC identity; a high-Oxphos, low-sympathetic niche may favor the activation of *Myl1*^+^ APCs over classical progenitors.

Our data support a model of reciprocal crosstalk among APC pools. The expression of inhibitory ligands, such as *Pdgfa, Notch3*, and *Jag1*, in *Myl1*^+^ APCs suggests that they may restrain adipogenesis in *Pdgfra*^+^/*Pdgfrb*^+^ APCs, coordinating a temporal handoff from neonatal to adult progenitor dominance. The compensatory activation of *Pdgfra*^+^ signatures when *Myl1*-lineage adipogenesis is blocked further supports a flexible, competitive hierarchy. Aging adds another layer of complexity. Our transcriptomic analyses reveal that aging-upregulated BAT gene sets are enriched for *Myl1*-lineage translational signatures, indicating that *Myl1*^+^ APCs may compensate for age-associated declines in other APC pools. *Myl1*^+^ APCs may therefore represent a stress-resistant progenitor population uniquely suited to preserving BAT function in late life. Whether their dominance reflects intrinsic increases in adipogenic potential or preferential survival of *Myl1*-derived adipocytes remains unresolved.

Collectively, our findings uncover a previously unrecognized progenitor-switching mechanism that governs adult BAT renewal and shapes brown adipocyte heterogeneity. We establish *Myl1*^+^ APCs as a specialized adult progenitor pool with distinct metabolic, molecular, and functional properties, whose PPARγ-dependent differentiation is indispensable for maintaining BAT homeostasis during aging. These insights highlight new opportunities to target progenitor identity and stromal crosstalk to preserve BAT function in metabolic disease.

## Methods

### Animals

All animal experiments were approved by the institutional animal care and use committee (IACUC) of the University of Minnesota and of University of Illinois Chicago and adhered to the NIH Guide for the Care and Use of Laboratory Animals. All the mice were group-housed in a light/dark cycle (12/12 h), temperature (21.5 ± 1.5 °C), and humidity-controlled (30-70%) room, and had free access to water and regular chow. *Myl1^Cre^* (Jax #024713), *Myl1^CreERT2^* (Jax #025670), *Pdgfra^CreERT2^* (Jax #032770). *Pparg^f/f^* (Jax #004584), *Rosa2^LtdTomato^*(Jax #007914), *Rosa26^LSL-mT/mG^*(Jax #007676), RiboTag (Jax #029977) mice were from Jackson Lab. The *Adipoq-LSL-DTR* (T058436) line was purchased from GemPharmatech.

For cold treatment, mice were housed in a temperature-controlled room (4°C) with free access to water. Core body temperature was measured using an electronic thermometer with an anal probe (Physitemp). Interscapular skin temperature was measured by anesthetizing mice with isoflurane and quickly capturing images using a FLIR C2 thermal camera. The average skin temperature within the interscapular region was analyzed using FLIR Thermal Studio.

For the glucose tolerance test, mice were fasted overnight for 16 hours and then intraperitoneally injected with 1.5 g/kg body weight of glucose. Blood glucose levels were measured using a glucometer at the indicated time points after injection.

### Tamoxifen preparation

Tamoxifen (Sigma, T5648) was dissolved at a concentration of 20 mg/mL in corn oil containing 10% ethanol. Briefly, tamoxifen powder was weighed into an amber tube, wetted with ethanol, and brought to volume with sterile corn oil. The suspension was vortexed thoroughly and then sonicated or incubated at 37 °C with intermittent mixing until it was fully dissolved. The stock solution was aliquoted into light-protected tubes and stored at −20 °C for up to several weeks. On the day of use, aliquots were equilibrated to room temperature and vortexed immediately before injection to ensure homogeneity.

### Timed breeding

For embryonic induction, timed matings were established by pairing one *Pdgfra^RFP^* male with two sexually mature *Pdgfra^RFP^* females in the late afternoon. The following morning, females were examined for the presence of a copulation plug by gently lifting the tail and visually inspecting the vaginal opening under bright light. Females with a visible vaginal plug were designated as embryonic day 0.5 (E0.5), separated into individual cages, and monitored daily for general health. Tamoxifen administration was scheduled based on this staging, with injections performed at the desired gestational days (e.g., E10.5, E14.5, or E17.5).

### Histology

Adipose tissues were fixed in a formalin or PFA solution at 4°C for 24 hours. Tissue embedding, sectioning, and hematoxylin and eosin (H&E) staining were performed at the Comparative Pathology Shared Resource of the University of Minnesota. For immunostaining, antigen retrieval was performed in Citric buffer using a 2100 Retriever (Aptum Biologics). After incubation with blocking buffer (3% BSA in PBS) for 1 hour, sections were incubated overnight at 4°C with the primary antibody in blocking buffer. For immunofluorescence, PBS-washed slides were incubated with a fluorescent secondary antibody at room temperature for 1 hour and then mounted with VECTASHIELD Antifade Mounting Medium with DAPI after three washes in PBS. A Keyence all-in-one fluorescence microscope was used for imaging.

### RT-qPCR

After weight measurement, BAT tissues were homogenized in TRIzol (Thermo Scientific) for RNA isolation, following the manufacturer’s protocol. RNA concentrations were measured with a NanoDrop spectrophotometer. Reverse transcription was performed with the iScript^™^ cDNA Synthesis Kit. Real-time RT-PCR was conducted using iTaq^™^ Universal SYBR^®^ Green Supermix and gene-specific primers on a Bio-Rad C1000 Thermal Cycler. Relative expression was normalized to the housekeeping *Rplp0* gene.

### SVF isolation, culture, and differentiation

BAT depots were collected and minced in 10 ml of digestion buffer (DMEM/F12 with 1mg/ml Collagenase I, 1% FBS, 1% HEPES, and 1% Pen-Strep). After shaking at 37°C and 100 rpm for 45 min, the digested tissues were filtered through 70-μm strainers and centrifuged at 1,500 rpm for 3 min. The pellets were resuspended in ACK buffer and put on ice for 5 min to remove red blood cells. The ACK buffer was neutralized with 5 ml of DMEM/F12 plus 10% FBS and removed after a 1,500-rpm centrifugation for 3 min. SVF cells were cultured with DMEM/F12 containing 20% FBS, 1% Pen-Strep, and 10 μg/ml Ciprofloxacin.

To test intrinsic adipogenic capacity, SVF cells were isolated from *Pdgfra^RFP^* mice pulsed at P3, P10, P30, or P60. SVF cultures were differentiated *in vitro* under standard adipogenic conditions. Briefly, confluent cells were induced with DMEM/F12 containing 10% FBS, 1x Pen-Strep, 20 nM insulin, 1 nM T3, 0.5 mM IBMX, 5 μM dexamethasone, and 125 μM indomethacin. Two days later, the cells were maintained in DMEM/F12 containing 10% FBS, 1x Pen-Strep, 20 nM insulin, and 1 nM T3. Medium was changed every other day until lipid droplets appeared.

### Ribosomal profiling and RNA-seq

RiboTag experiments were carried out following an established protocol^59^. Briefly, iBAT samples from *RiboTag^Myl1-Cre^* and *RiboTag^Ucp1-Cre^* mice were harvested and lysed in ice-cold homogenization buffer with Dounce tissue grinders. Lysate was cleared by centrifugation for 10 min at 10,000 g, 4°C. A small aliquot was stored at −80°C for subsequent Input analysis. The remaining lysate was incubated with anti-HA antibody and protein A/G beads for overnight at 4°C. Next day, beads were centrifuged and washed with high salt buffer for 3 times. RNA was extracted using the Qiangen RNeasy mini kit as described in the instructions. RNA quality was determined by an Agilent 2100 Bioanalyzer. Total RNA from Input and IP samples were subjected to dual-indexed TruSeq stranded mRNA library preparation and sequenced on a NovaSeq 2x150-bp run at the University of Minnesota Genomics Center. A pipeline developed and maintained by the Research Informatics Solutions (RIS) group at the University of Minnesota Informatics Institute (UMII) was used for RNA-seq analysis. Differentially expressed genes were detected using DEseq2 or EdgeR.

To compare BAT transcriptome of young and old mice, two publicly available bulk RNA-seq datasets (GSE135391 and GSE141252) were used. Raw gene counts from GSE135391 (including 3- and 24-month-old mice at RT) were extracted from ARCHS^4^ (All RNA-seq and ChIP-seq sample and signature search)^60^, and merged with those from GSE141252 (BAT from 4 and 24 month old mice) using R. Differentially expressed genes (fold change > 2 and adjust P value < 0.1) were determined with iDEP (integrated Differential Expression & Pathway analysis, v 2.20.5) with the correction for batch effect^61^. Significantly upregulated and downregulated gene sets were created (Table. S7) for Gene Set Enrichment Analysis (GSEA, v 4.4.0)^62^.

### Analysis of scRNA-seq and snRNA-seq

Seurat (v5.0.0) on RStudio (2023.12.0, R version 4.3.2) was used for integrative scRNA-seq analysis of GSE207706 (iBAT from mice housed at RT or cold 4 days)^24^, GSE160585 (iBAT from mice housed TN for 1 week, RT, or cold for 2 or 7 days)^31^, and GSM5068995 (paBAT from adult mice)^21^. Individual samples were filtered (> 100 features, < 4, 000 features and < 10% of genes mapped to mitochondria) and normalized with the SCTransform function. Integration was performed with the FindIntegrationAnchors function with “rpca” reduction and the IntegrateData function with “SCT” as the normalization method. After finding neighbors, clusters, and markers, the subset of lineage-negative cells was created and re-clustered (dims = 1:30, resolution = 0.1). UMAP and tSNE were then used for two-dimensional visualization of the resulting cluster. Marker genes were identified using the FindAllMarkers function (only.pos = TRUE, min.pct = 0.25, logfc.threshold = 0.25). Violin plots, dot plots, heatmaps, and individual tSNE and UMAP plots for the given genes were generated by using the VlnPlot, DotPlot, DoHeatmap, and FeaturePlot functions, respectively.

snRNA-seq data of freshly isolated mouse brown adipocytes (GSM3567479) was ^ltered with > 200 features, < 3, 000 features and < 75% of genes mapped to mitochondria. Seurat was used to NormalizeData, ScaleData, FindVariableFeatures, RunPCA, FindNeighbors, FindClusters (resolution = 0.2), and RunUMAP (dims = 1:30). The subset of brown adipocytes was clustered again to FindAllMarkers. *RiboTag^Myl1-Cre^* ribosome-enriched and -depleted genes were visualized in the UMAP space with the AddModuleScore and FeaturePlot functions.

### Quantification and statistical analysis

Results are shown as mean ± SEM or ± SD. N values (biological replicates) and statistical analysis methods are described in the figure legends. The statistical comparisons were carried out using two-tailed Student’s t-test and one-way or two-way ANOVA with indicated post hoc tests with Prism (Graphpad). Differences were considered significant when p < 0.05. *, p < 0.05; **, p < 0.01; ***, p < 0.001.

## Supplementary Material

This is a list of supplementary files associated with this preprint. Click to download.

• FiguresSv4.docx
